# Immune microenvironment regulation and clinical immunotherapy strategies of metastatic liver cancer

**DOI:** 10.3389/fimmu.2025.1633315

**Published:** 2025-09-09

**Authors:** Dan Liu, Mingzhu Li, Ying Liang, Fang Xu, Runtian Li, Yang Sun

**Affiliations:** Department of Biology, College of Basic Medicine, Heilongjiang University of Chinese Medicine, Harbin, China

**Keywords:** metastatic liver cancer, tumor microenvironment, innate immune cells, adaptive immunity, Kupffer cells, immunotherapy

## Abstract

Metastatic liver cancer (MLC) remains a leading cause of cancer-related mortality due to the liver’s unique immunotolerant microenvironment and high vascularization. Key mechanisms involve KC-mediated fibronectin deposition, neutrophil extracellular traps (NETs), and MDSC-driven T-cell exhaustion. Clinically, therapeutic strategies targeting the tumor microenvironment (TME) such as CSF1R inhibition, CCR2/CCR5 blockade, and CD40 agonism show promise in preclinical and early-phase trials, especially when combined with immunotherapy. However, challenges remain in overcoming systemic immunosuppression. This review summarizes the dual roles of hepatic immune cells including Kupffer cells (KCs), neutrophils, and myeloid-derived suppressor cells (MDSCs) in either suppressing or promoting metastatic colonization. We elucidate how the liver’s immunological balance, governed by innate and adaptive responses, shifts toward immunosuppression during metastasis, fostering a pro-tumor niche. This synthesis of immunological insights underscores the potential of TME-modulating therapies to improve outcomes in MLC.

## 1 Introduction

Metastatic liver cancer (MLC) is a secondary malignancy arising from both gastrointestinal and non-gastrointestinal primary tumors. Gastrointestinal-derived metastases, though originating in the digestive tract, frequently disseminate to distant organs via hematogenous routes (1, 2). Due to the liver’s unique anatomical position and portal circulation, it serves as the predominant site for metastatic seeding in gastrointestinal cancers (3). MLC significantly contributes to cancer-related mortality (4, 5), with hepatic metastases conferring poor prognoses across malignancies, including breast, renal, and lung cancers. Notably, 25% of newly diagnosed CRC patients and 40%–50% with advanced CRC develop liver metastases (6).

The liver’s high metastatic susceptibility stems from its dual blood supply and hemodynamic architecture, which promote tumor cell homing (7). Beyond vascular mechanisms, the hepatic microenvironment critically supports metastatic colonization, making therapeutic targeting of the tumor microenvironment (TME) a key research focus (8, 9). This review summarizes the roles of hepatic immune cells, including Kupffer cells (KCs), neutrophils, and myeloid-derived suppressor cells (MDSCs), in either suppressing or promoting metastatic colonization. By synthesizing hepatic immune responses, microenvironmental dynamics, and clinical evidence, we explore TME modulation as a potential strategy for MLC prevention and therapy.

## 2 The unique hepatic immune microenvironment dictates the fate of metastatic cancer cells

### 2.1 Innate immune responses in the liver

The liver’s immune system is uniquely adapted to maintain tolerance to portal vein-derived antigens under homeostasis (10, 11), yet it can mount robust immune responses against acute threats like metastatic invasion (12). Upon entering the liver, cancer cells encounter a specialized cellular milieu that orchestrates antigen presentation, pathogen recognition, and targeted elimination (13). Natural killer (NK) cells dominate the hepatic lymphocyte population (14), playing a pivotal role in immune surveillance. Unlike adaptive immune cells, NK cells detect targets lacking MHC-I—a common evasion strategy employed by tumors and pathogens (15, 16). The liver also harbors invariant natural killer T (iNKT) cells, a unique subset derived from thymic CD4^-^CD8^-^ precursors that mature into CD4^+^CD8^+^ effectors (17, 18). These cells express chemokine receptors (CCR5/CXCR3) and patrol liver sinusoids via CD1d-dependent interactions with liver sinusoidal endothelial cells (LSECs) and macrophages, enabling rapid anti-tumor responses (19, 20). However, during metastatic progression, iNKT cells exhibit functional impairment (21). Studies have shown that tumor-induced immunosuppressive cytokines, such as IL - 10 and TGF-β, downregulate their cytotoxic capacity and IFN-γ production. Additionally, the altered expression of CD1d and co-stimulatory molecules on antigen-presenting cells in the metastatic liver microenvironment diminishes iNKT cell activation (22, 23). This dysfunction facilitates immune evasion by metastatic cells and contributes to the establishment of an immunosuppressive niche. Beyond the resident Kupffer cells (KCs), the liver recruits CCR2^+^Ly6C^+^ monocytes from the bone marrow during inflammation (24). These monocytes are significantly upregulated in pathological states of the liver, and studies in CCR2^-/-^ mice have demonstrated that their absence mitigates hepatic inflammation (25). Neutrophils are also actively recruited to sites of hepatic inflammation (26). These cells express adhesion molecules such as CD44, Siglec-9 (27), Siglec-10 (28), and very late antigen-4 (VLA - 4) (29), which mediate their adherence to vascular adhesion molecules on LSECs.

### 2.2 Adaptive immune responses in the liver

The liver maintains a delicate immunological equilibrium, balancing tolerance to dietary and microbial antigens with defense against pathogens and malignancies. This balance is orchestrated by hepatic antigen-presenting cells (APCs), which under steady-state conditions drive tolerogenic T cell responses, facilitating transplantation tolerance and chronic viral infections such as HBV and HCV (30, 31). LSECs function as tolerogenic APCs by expressing PD-L1 and inducing T cell exhaustion, suppressing Th1 differentiation while favoring IL - 4^+^ Th2 polarization. Meanwhile, KCs that resident liver macrophages exhibit low MHC II and co-stimulatory molecule (B7 - 1/2) expression, thereby limiting T cell activation and fostering immunosuppression via PD-L1 and cytokine secretion (32). However, upon stimulation with inflammatory cues such as TLR ligands, cytokines, and PolyI:C, KCs transition to an immunogenic phenotype, upregulating MHC II and activating iNKT cells, suggesting the existence of functionally distinct KC subsets (33, 34).

**Figure 1:**
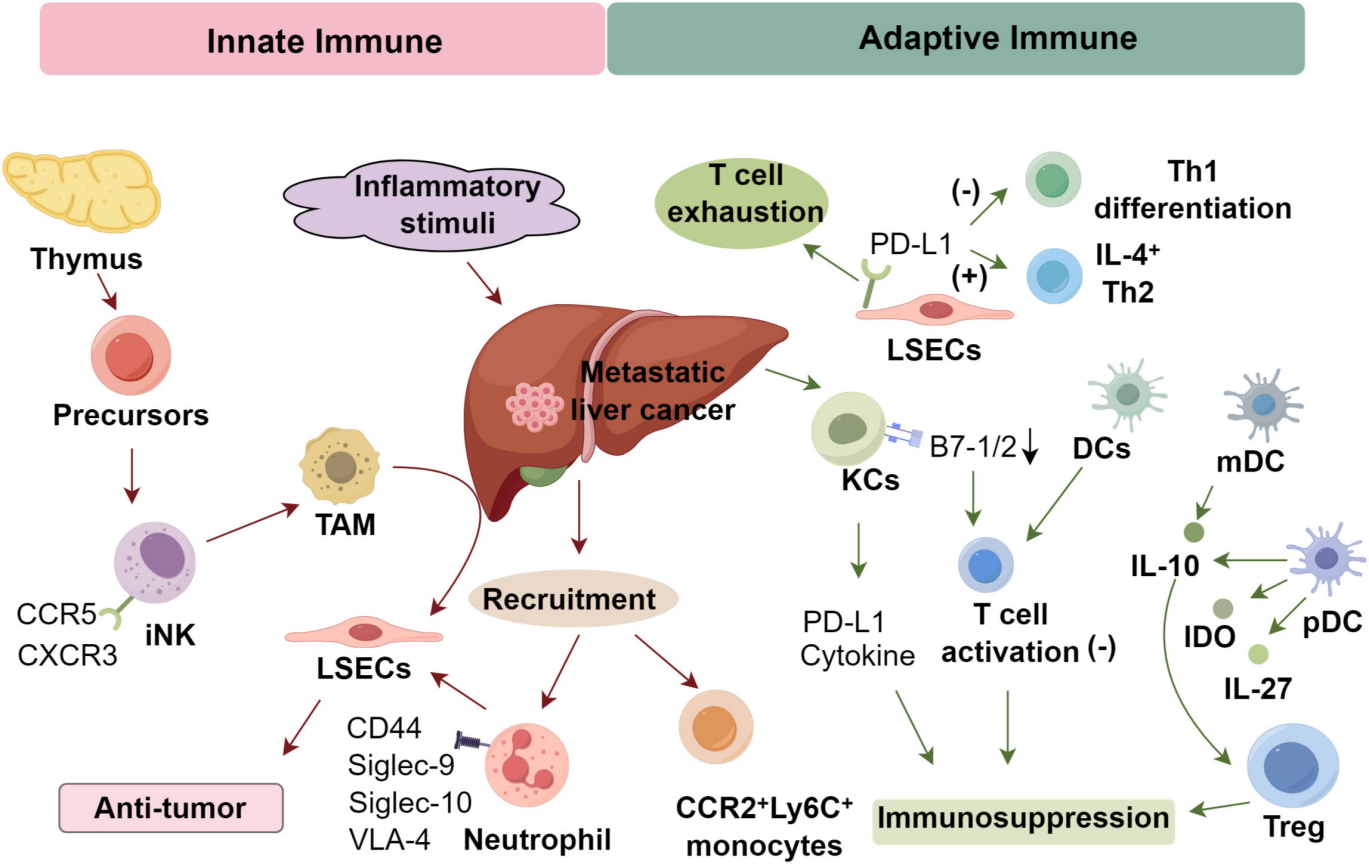
Immune microenvironment in metastatic liver cancer.

Hepatic dendritic cells (DCs), including CD11b^+^, CD11c^high^, CD1c^+^, myeloid DCs (mDCs), and plasmacytoid DCs (pDCs), generally suppress T cell activation. In mice, subsets like CD11c^+^CD8^+^ and CD11c^+^NK1.1^+^ DCs also exist but remain poorly characterized (32, 35). Hepatic mDCs and pDCs secrete IL - 10 and are regulated by macrophage colony-stimulating factor (M-CSF), which enhances IL - 10 while suppressing IL - 12 (36). pDCs also produce IL - 27 and IDO, promoting Treg expansion and immunosuppression (37, 38). Their low Delta4/Jagged1 Notch ligand ratio biases toward Th2 differentiation and CD4^+^ T cell apoptosis, reinforced by Treg-mediated inhibition and PD-L1–PD-1 signaling (39). Lipid-poor DCs tend to be tolerogenic; however, CD11c^+^CD8^+^ DCs elicit strong Th1 responses via IL - 12 and TNF-α, while CD11c^+^NK1.1^+^ DCs exhibit cytolytic activity and stimulate T cell immunity. Hepatocytes also present antigens via MHC II, contributing to antiviral defense, though their antitumor role remains uncertain (40). Hepatic stellate cells (HSCs), residing in the space of Disse, act as APCs and play a significant immunomodulatory role in the hepatic immune microenvironment. They express immune checkpoint molecules such as PD-L1 and secrete immunosuppressive mediators including IL - 6, IL - 10, and TGF-β, which collectively promote the expansion of regulatory T cells (Tregs) and contribute to the exhaustion of effector T cells (41–44). In addition, HSCs can express indoleamine 2,3-dioxygenase (IDO), further suppressing T cell proliferation and cytokine production through tryptophan depletion and kynurenine accumulation, thereby reinforcing immune tolerance (45, 46). Through CD44-dependent signaling, HSCs also convert recruited monocytes into myeloid-derived suppressor cells (MDSCs), exacerbating local immunosuppression and facilitating metastatic colonization (47). Overall, hepatic antigen presentation often favors immunosuppression, shaped by the dynamic interplay of tolerogenic and immunogenic signals within the hepatic microenvironment (**Figure 1**).

## 3 Pro-metastatic tumor microenvironment of the liver

### 3.1 Role of Kupffer cells in cancer cell metastasis

KCs, the liver’s resident macrophages, regulate cholesterol metabolism, pathogen clearance, and immune responses (48, 49). Originating from yolk sac-derived progenitors, KCs are replenished by bone marrow-derived precursors during hepatic injury or infection (50). They detect pathogens via diverse receptors, secreting cytokines to initiate innate immune responses (33, 51). KCs facilitate metastasis by forming a pre-metastatic niche. In pancreatic cancer, KCs internalize tumor-derived exosomes containing macrophage migration inhibitory factor, triggering TGF-β secretion and hepatic stellate cell (HSC)-mediated fibronectin production, promoting metastatic cell adhesion (52). Circulating tumor cells bind fibronectin via Talin-1, enhancing colonization (53). KCs exhibit dual roles in metastasis: early cytolysis versus later pro-tumor support. Depleting KCs increases metastatic burden, suggesting initial tumoricidal activity (54–56). KCs phagocytose tumor cells via Dectin-2 or other receptors, though post-internalization viability remains unclear (57). Cytotoxic NO, NK cell activation, and TNF-α secretion further limit early metastasis (58, 59). However, KC-derived cytokines may aid surviving tumor cells post-extravasation. Myeloid cell recruitment complicates KC-specific roles, as depletion strategies often affect other phagocytes. Thus, early-phase studies are critical to delineate KC contributions (57).

### 3.2 Role of neutrophils in cancer cell metastasis

One of the earliest pathological responses to hepatic cancer cell infiltration is neutrophil recruitment (60, 61). Normally, neutrophils migrate to inflamed sites by rolling along vascular endothelium via low-affinity binding to P-/E-selectins, followed by integrin-mediated firm adhesion and arrest, primarily in post-sinusoidal venules, though CD44-hyaluronan interactions are not involved in hepatic sinusoids (62). Tumor-associated neutrophils (TANs), like Kupffer cells, exhibit dual pro- and anti-metastatic roles (63). In colorectal liver metastases (CRLM), neutrophils promote progression, with elevated neutrophil-to-lymphocyte ratio (NLR) correlating with worse outcomes, though absolute neutrophil counts yield conflicting data (64). Higher neutrophil numbers generally predict poorer prognosis (65). Experimental models reveal neutrophils facilitate multiple metastatic steps (66). In pancreatic cancer GEMMs, they aid pre-metastatic niche formation, while neutrophil extracellular traps (NETs) enhance early cancer cell retention by physically ensnaring circulating tumor cells within the hepatic vasculature. Mechanistically, NETs release high-mobility group box 1 (HMGB1), which activates TLR9 signaling in tumor cells, promoting their proliferation and metastatic competency (67, 68). Moreover, NET-associated proteases such as neutrophil elastase and matrix metalloproteinase 9 (MMP9) degrade extracellular matrix (ECM) components, thereby facilitating tissue invasion and the establishment of a pro-metastatic niche (69, 70). Post-colonization, neutrophils accelerate growth via fibroblast growth factor 2 (FGF2), with FGF2 inhibition reducing metastatic burden (71). Neutrophils also modulate CD8^+^ T cell responses in metastatic liver cancer (MLC) and exhibit heterogeneous N1/N2 phenotypes regulated by TGF-β and IGF1, influencing pro- or anti-tumor effects (72, 73). Notably, transforming growth factor-β (TGF-β), secreted by metastatic tumor cells and Kupffer cells within the liver, is a key immunosuppressive cytokine that drives the polarization of neutrophils toward a pro-tumor phenotype (74, 75). TGF-β signaling inhibits neutrophil cytotoxicity and reactive oxygen species (ROS) production, while promoting the expression of matrix metalloproteinases MMP - 9 and vascular endothelial growth factor (VEGF) (76–79), thereby enhancing tumor angiogenesis and extracellular matrix remodeling. Moreover, TGF-β suppresses neutrophil-mediated stimulation of CD8^+^ T cell responses, further contributing to immune evasion in the metastatic tumor microenvironment (80, 81). In addition, IGF1 has been shown to further modulate the polarization of neutrophils, especially in liver metastasis, acting as a significant driver of the neutrophil polarization in this organ (82, 83). Thus, neutrophils drive metastasis at multiple stages, with TGF-β and IGF1 synergistically enhancing their pro-metastatic functions in liver metastases.

### 3.3 Recruitment of monocytes/macrophages and myeloid-derived suppressor cells to metastatic sites

Bone marrow-derived cells, including monocytic MDSCs (M-MDSCs) and granulocytic MDSCs (G-MDSCs), are recruited to the liver, facilitating metastatic expansion (84–86). In colorectal liver metastasis, macrophage infiltration is predominantly mediated by CCL9 and CCL15, which recruit CCR1^+^ macrophages, whereas granulocytic MDSCs are recruited via CCR2 (87, 88). Tumor-associated macrophages (TAMs) promote MLC growth, and their depletion reduces metastatic foci. Chemotactic factors drive macrophage recruitment, and blocking these signals attenuates metastasis. Kitamura et al. (89) identified CCL9 and CCL15 as CRC-secreted chemokines recruiting CCR1^+^ macrophages; CCR1 inhibition impairs infiltration and suppresses metastasis.

TAMs support metastasis via immune-dependent and independent mechanisms (90). They promote angiogenesis via VEGFR1, responding to tumor-derived VEGF and complement factors. CRC cells produce C5a, binding macrophage C5aR to enhance recruitment and M2 polarization, fostering metastasis. Conversely, C5aR ablation reduces M2 accumulation and metastatic burden (91, 92). In pancreatic cancer, macrophages secrete granulin, activating hepatic stellate cells (HSCs) to produce ECM and support metastasis. Lim et al. (93) found macrophage depletion upregulated S100A8/S100A9 and downregulated ANGPTL7 in cancer cells, altering metastatic potential. S100A8/A9 silencing reduced MLC formation, while ANGPTL7 overexpression suppressed it, indicating macrophage-mediated tumor reprogramming. Hypoxia in metastases enhances macrophage pro-metastatic functions (94). In HCC, hypoxia and necrosis induce HIF - 1α and TLR4 in macrophages, boosting IL - 1β production, ECM deposition, and metastasis (95). Cirrhotic mice show increased metastasis with reduced NO, while high-fat diet (HFD)-fed mice exhibit non-alcoholic fatty liver disease (NAFLD)-linked metastasis and M2 macrophage infiltration. NLRC4 deficiency abrogates HFD effects, and NAFLD-associated IL - 1β promotes HCC metastasis (96). Distinguishing resident from monocyte-derived macrophages is critical for therapy (97). Tumor secretomes homogenize macrophage populations toward pro-tumor phenotypes (98, 99), though ontogeny influences function, as CSF1R blockade affects brain microglia differently (100).

MDSCs suppress innate and adaptive immunity in metastasis (101, 102). M-MDSCs are often associated with immunosuppressive functions and T-cell inhibition, primarily through the production of arginase-1 and IDO, which impair T-cell function and promote Treg expansion (103). These M-MDSCs are frequently localized at the tumor stroma or the tumor periphery, where they interact with KCs and other stromal cells to suppress effector immune responses (104, 105). In contrast, G-MDSCs, which are typically characterized by the expression of Ly6G, mediate their immunosuppressive effects through neutrophil extracellular trap (NET) formation (106, 107). This mechanism facilitates the entrapment of circulating tumor cells in the hepatic vasculature and promotes tumor cell adhesion. Additionally, the release of HMGB1 by NETs activates TLR9 signaling in tumor cells, enhancing their metastatic potential (108). G-MDSCs are predominantly localized to microvascular niches within the hepatic sinusoids during early metastatic colonization, where they exert their pro-metastatic functions by altering the extracellular matrix (ECM) and promoting angiogenesis (109, 110). Recruited via LSEC/KC/HSC chemokines, their hepatic accumulation in female mice is estrogen-dependent and TNFR2-mediated (111). Tumor-derived VEGF induces macrophage CXCL1, recruiting MDSCs (112). STAT3 activation via sphingosine-1-phosphate receptor 1 (S1PR1) drives IL - 6-mediated MDSC accumulation (113), though signals preventing their maturation remain unclear (114). MDSCs are identified by CD11b, Ly6G, and Ly6C, but marker overlap with TAMs/TANs complicates characterization (115) (**Table 1**).

**Table 1:** Key immune cell populations in the hepatic metastatic niche and their functional roles.

Cell Type	Subsets	Pro-Metastatic Mechanisms	Anti-Metastatic Mechanisms	Clinical Targeting Strategies
Kupffer Cells (KCs)	Resident (yolk sac-derived), BM-derived	Pre-metastatic niche formation via TGF-β/fibronectin; cytokine support post-extravasation.	Early-phase tumor phagocytosis (Dectin-2), NO/TNF-α secretion, NK cell activation	CSF1R inhibitors (pexidartinib), CD40 agonists
Neutrophils	N1 (anti-tumor), N2 (pro-tumor)	NETs enhance colonization; FGF2-driven growth; NLR correlates with poor prognosis	Limited direct cytotoxicity; N1 phenotype inhibits metastasis under TGF-β blockade	CXCR2/CXCR4 inhibition (BL - 8040), NET disruption
Monocytes /Macrophages	TAMs (M1/M2), CCR2^+^Ly6C^+^ inflammatory monocytes	CCL9/CCL15-CCR1 recruitment; VEGFR1 angiogenesis; C5aR-mediated M2 polarization	M1 phenotype exerts phagocytic activity; TLR activation may restore antitumor function	CCR2/CCR5 antagonists (maraviroc), CCL2/CXCL12 axis blockade
MDSCs	PMN-MDSCs (CD11b^+^Ly6G^+^), M-MDSCs (CD11b^+^Ly6C^+^)	STAT3/IL-6-driven expansion; S1PR1-mediated immunosuppression; estrogen-dependent recruitment	None identified in metastasis	CXCR4 inhibitors, PD - 1/CTLA-4 combo therapy
iNKT Cells	CD4^+^CD8^+^ double-positive	Rarely pro-tumor; may promote fibrosis via HSC interaction	CD1d-dependent cytotoxicity; IFN-γ secretion against MHC-I^-^ targets	α-GalCer analogs to activate iNKT cells (phase I/II trials)

### 3.4 Metabolic constraints of the tumor microenvironment impair immune effector functions

The immunosuppressive TME in metastatic liver cancer is not only shaped by cellular interactions but also by profound metabolic reprogramming that impairs cytotoxic immune responses (116, 117). Tumor cells consume glucose at a high rate through aerobic glycolysis (the Warburg effect), leading to glucose depletion in the hepatic niche (118, 119). Since both NK cells and cytotoxic CD8^+^ T cells rely on glucose-driven oxidative phosphorylation and aerobic glycolysis to sustain their effector functions, nutrient scarcity results in cellular exhaustion and reduced cytokine secretion (IFN-γ, TNF-α) (120, 121). Additionally, lactate—a byproduct of tumor glycolysis—is exported via MCT4 into the extracellular space (122, 123). Its accumulation acidifies the TME and is taken up by immune cells, causing intracellular acidosis that disrupts signaling pathways such as NFAT and mTOR, thereby suppressing IFN-γ production in NK and T cells (124). Moreover, hypoxia, a hallmark of the liver metastatic TME, stabilizes HIF - 1α in NK and T cells, shifting their metabolism toward anaerobic pathways and impairing mitochondrial function, proliferation, and cytolytic activity (120, 125). Collectively, these metabolic stressors within the TME undermine the survival and effector potency of immune cells, further favoring metastatic colonization.

## 4 Clinical trials targeting TAMs and MDSCs

TAMs and MDSCs critically sustain the immunotolerant milieu of metastatic liver cancer (MLC), making them prime therapeutic targets (126, 127). The CSF1/CSF1R axis regulates macrophage differentiation, recruitment, and survival. CSF1R inhibitors reduce CD68^+^/CD163^+^ macrophage infiltration in normal liver tissue. In colorectal cancer (CRC) models, CSF1R blockade elevates cytotoxic T cells while suppressing FoxP3^+^ Tregs (128). Though limited as monotherapy (129), CSF1R inhibition synergizes with PD - 1/PD-L1 inhibitors or chemotherapy. A phase I trial (NCT02777710) combining durvalumab (PD-L1 inhibitor) and pexidartinib (CSF1R inhibitor) in advanced CRC/pancreatic cancer showed 21% achieving stable disease ≥2 months (130). This limited efficacy of CSF1R blockade as monotherapy may stem from compensatory mechanisms that sustain TAM survival and function (131, 132). In particular, GM-CSF and G-CSF signaling pathways can support macrophage viability and polarization in the absence of CSF1R signaling, enabling the persistence of pro-tumoral macrophage populations despite CSF1R inhibition (133, 134). Additionally, tumors may circumvent CSF1R blockade by recruiting alternative immunosuppressive cell types, including tumor-associated neutrophils (TANs), MDSCs, and tolerogenic dendritic cells, which collectively reinforce an immunosuppressive microenvironment (135). These compensatory pathways highlight the need for combination therapies that simultaneously target multiple immunoregulatory axes within the tumor microenvironment.

Disrupting TAM/MDSC recruitment offers another strategy. CCL2, CXCL12, and CCL5 mediate hepatic infiltration by these cells (136, 137). In CRC models, CCL2 correlates with MLC progression. CCR2 knockout mice exhibit reduced TAMs, increased CD8^+^/CD4^+^ T cells, and improved survival (138). Clinically, the CCR2 antagonist CCX872 plus FOLFIRINOX improved survival in metastatic pancreatic cancer, with ~33% alive at 18 months (139). An ongoing trial (NCT03184870) is testing the CCR2/CCR5 antagonist BMS - 813160 with chemo/immunotherapy in metastatic pancreatic/CRC. The CXCL12/CXCR4 axis also recruits immunosuppressive cells to the liver (140). In CRC models, CXCR4 inhibition reduced MLC/MDSC accumulation (141) and enhanced PD - 1 blockade efficacy, elevating CD8^+^ T cell/Treg ratios and tumor regression (142). A trial combining the CXCR4 inhibitor BL - 8040 with FOLFIRI/pembrolizumab in refractory pancreatic cancer yielded 4 partial responses among 15 patients (143). Further trials (NCT02907099) will clarify its role in MLC.

The CCL5/CCR5 axis drives metastasis by mobilizing MDSCs and polarizing M2 macrophages (144, 145). In CRLM, CCR5^+^ tumors exhibit elevated Treg: CTL ratios and PD - 1/CTLA-4 (146). Preclinical data show CCL5 boosts TAM-derived MMPs, accelerating progression, while maraviroc (CCR5 inhibitor) reprograms TAMs to an antitumoral phenotype. A phase I trial (MARACON) in CCR5^+^ mCRC saw 3/11 patients respond post-chemotherapy (147). Ongoing studies (NCT03274804, NCT03631407) are testing CCR5/PD-1 co-blockade in MSS mCRC. Reprogramming TAMs toward antitumor states is another approach. CD47-SIRPα signaling inhibits macrophage phagocytosis, and CD47 upregulation helps tumors evade immunity (148). In models, CD47 inhibition reduced MLC (149), prompting phase I trials of CD47 blockers alone (NCT04257617, NCT03763149) or combined (NCT02953782). CD40 agonists activate macrophages via T cell-dependent/independent pathways, inducing IFN production and ECM remodeling (150). A phase Ib trial combining gemcitabine/nab-paclitaxel/CD40 agonist ± nivolumab in metastatic pancreatic cancer achieved a 58% response rate (151). Other agents promoting M1 polarization include TLR agonists, PI3Kγ inhibitors, and HDAC inhibitors (152–154). The liver’s immunotolerant microenvironment is shaped by bone marrow/lymphoid-derived immunosuppressive cells, fostering metastasis and impairing systemic immunity. Overcoming this requires multimodal strategies, with current research focusing on enhancing immunotherapy efficacy in MLC.

## 5 Conclusion

Metastatic liver cancer (MLC) represents a formidable clinical challenge, where the liver’s unique immunotolerant microenvironment actively facilitates tumor colonization and progression. Our review highlights the dual roles of hepatic immune cells - initially serving as a defense barrier but ultimately being co-opted to support metastatic growth through multiple mechanisms. Kupffer cells transition from tumoricidal effectors to pro-metastatic facilitators, while recruited neutrophils and MDSCs establish immunosuppressive networks via NETosis, cytokine secretion, and metabolic competition. These cellular interactions create a self-reinforcing niche that promotes immune evasion and treatment resistance.

To overcome these challenges, future therapeutic strategies must integrate TME-modulating agents with immunotherapy and chemotherapy, guided by comprehensive immune profiling. Emphasis should be placed on identifying predictive biomarkers and understanding spatiotemporal immune evolution during metastasis. By elucidating the complex immunobiology of liver metastasis, this review highlights the potential of combinatorial approaches to transform MLC treatment and improve patient outcomes.
